# Effects of lubrication on air-sealing performance of a pediatric cuffed tracheal tube

**DOI:** 10.1186/s12871-017-0416-1

**Published:** 2017-09-19

**Authors:** Hiroko Nishioka, Yutaka Usuda, Go Hirabayashi, Koichi Maruyama, Tomio Andoh

**Affiliations:** 1 Department of Anesthesiology, Mizonokuchi Hospital, Teikyo University School of Medicine, 3-8-3 Mizonokuchi, Takatsu-ku, Kawasaki, 213-8507 Japan; 2 Department of Anesthesiology, Saitama Medical University International Medical Center, 1397-1 Yamane, Hidaka City, Saitama, 350-1298 Japan

**Keywords:** Air leakage, K-Y jelly, Tracheal tube

## Abstract

**Background:**

Lubrication of cuffed tracheal tubes (CTTs) reduces liquid leakage. However, it is not clear how cuff lubrication influences air leakage. We aimed to test the hypothesis that pretreatment with K-Y jelly, a water-soluble lubricant, would improve the air-sealing performance of pediatric CTTs in a model study.

**Methods:**

We placed Parker Flex-Tip™ CTT with 4.0- and 5.0-mm internal diameter (ID) into a tracheal model with 9- and 12-mm ID. The tracheal model was connected to a test lung ventilated in pressure control mode. We compared three cuff lubrication conditions: none (N), water (W), and K-Y jelly (KY). We measured the leak airway pressure (LAWP), defined as the lowest peak airway pressure (PAWP) at which leakage was detected, with the fixed cuff pressure (CP) at 20 cmH_2_O and varied PAWP. We also measured the leak CP (LCP), defined as the highest CP at which leakage was detected, with fixed PAWP at 25 cmH_2_O and varied CP. We confirmed air leakage when an apparent elevation of oxygen concentration was detected above the cuff after changing the inspiratory gas from air to oxygen.

**Results:**

For both 4.0-mm ID and 5.0-mm ID endotracheal tubes, the KY group showed significantly higher LAWP and lower LCP than the other two groups. For the 4.0-mm ID, median values and ranges of LAWP and LCP were K-Y group: 25 (25) and 15 (15); N group: 5 (5) and 35 (35): and W group: 5 (5) and 35 (15–35) cmH_2_O. For the 5.0-mm ID, median values and ranges of LAWP and LCP were K-Y group: 25 (15–25) and 15 (15–35); N group: 5 (5) and 35 (35); and W group: 5 (5) and 35 (15–35) cmH_2_O. Water application did not change these outcomes compared with the N group.

**Conclusion:**

Pre-treatment of the cuff with K-Y jelly significantly improved the air-sealing performance of a pediatric CTT in our model study.

## Background

It is well known that the materials and shapes of endotracheal tube cuffs influence their air-sealing properties. [1, 2] For example, Microcuff pediatric tracheal tubes have been shown to exhibit better air-sealing performance than conventional cuffs. [1] It is also known that lubrication of cuffed tracheal tubes reduces liquid leakage in adult cuffed tubes in a bench-top study and clinical studies. [3–5] However, it is not clear how pre-treatment of the cuff with lubricants influences air leakage. It is likely that longitudinal folds formed on the cuff wall serve as channels for both air and liquid to pass through and that lubrication may fill these channels and similarly reduce both liquid and air leakage. [6] However, there is a large difference in viscosity between air and liquid. The viscosity of oxygen is approximately 500 times lower than that of airway secretions. [7, 8] In addition, the pressure gradients across the channels are different for the two types of leakage. [9, 10] Fluid leakage seemed to be regulated by the pressure difference between the hydrostatic pressure of fluid accumulated on the cuff and the end-expiratory airway pressure, [10] whereas air leakage may be influenced by the peak airway pressure (PAWP). [9] These physical and rheological factors would greatly influence the nature and magnitude of the leakage. If we assume that leakage has properties of laminal flow, the leak flow would be correlated with the pressure gradient and inversely correlated with viscosity. [11] Using a tracheal model, we aimed to test the hypothesis that pretreatment with K-Y jelly, a water soluble lubricant, would improve the air-sealing performance of pediatric cuffed tracheal tubes.

## Methods

**Fig. 1:**
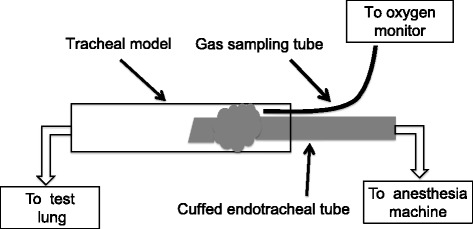
Experimental setup for the in vitro assessment of air leakage A tracheal model was intubated with a pediatric cuffed endotracheal tube that was connected to a test lung. An anesthesia machine was connected to the endotracheal tube to ventilate the test lung. We placed a gas sampling tube just above the cuff to confirm air leakage by detecting an increase in oxygen concentration after changing the inspiratory gas from air to oxygen.

We placed Parker Flex-Tip™ tracheal tubes of the high-volume low-pressure cuffed type with an internal diameter (ID) of 5.0 mm (Parker tube; Parker Medical, Highlands Ranch, CO, USA, International product number: I-PFHV-50) into a tracheal model consisting of an acrylic cylinder with ID of 12 mm to simulate an approximately 8-year-old patient. [12, 13] We also used Parker Flex-Tip™ tracheal tubes of the preformed oral cuffed type with ID of 4.0 mm (International product number: I-PFOC-40) and an acrylic tracheal model with ID of 9 mm to simulate an approximately 4-year-old patient. The cuffs of the both tracheal tubes are made of polyvinyl chloride (PVC) and have similar cylindrical shapes. We chose Parker Flex-Tip™ tracheal tubes as representative examples of tubes with cylindrical shaped PVC cuffs. The tracheal model was connected to a test lung, and the tracheal tube was connected to an Avance Carestation (GE HealthCare, Fairfield, CT, USA) through a respiratory circuit. The test lung was ventilated with the pressure control mode at an inspiratory to expiratory time ratio of 1:2 and frequency of 15 breaths/min. We compared three lubrication conditions: None, Water, and K-Y jelly (KY; Johnson & Johnson, New Brunswick, NJ, USA). We weighed the jelly and dipped the cuff into the jelly to standardize the lubrication condition. The amount of K-Y jelly used was 1.5 g for ID 5.0-mm and 1.2 g for ID 4.0-mm tracheal tubes. As depicted in Fig. 1, we placed a gas sampling tube just above the cuff to measure oxygen concentration. We confirmed an air leak when an apparent elevation of O_2_ concentration was detected within 2 min after switching the inspiratory gas from air to pure oxygen. Oxygen concentration was measured with an anesthetic gas analyzer (M1019A Intelliview, Philips, Andover, MA, USA). The duration of the experiment for each condition was 10 min; we waited during a 5-min stabilization period and then observed the O_2_ concentration for 5 min after changing the inspiratory gas.

We performed two series of measurements by testing 5 new 4.0-mm ID tracheal tubes and 10 new 5.0-mm ID tracheal tubes. During experiment (A), the cuff pressure (CP) was maintained at 20 cmH_2_O while the PAWP was increased from 5 to 25 cmH_2_O in increments of 5 cmH_2_O. We defined the leak airway pressure (LAWP) to be the lowest PAWP at which leakage was detected. When no leakage was detected, the highest PAWP tested was considered the LAWP. The airway pressure was based on the settings of the built-in ventilator of the Avance Carestation, and the CP was measured with a cuff manometer (VBM Medizintechnik GmbH, Sulz am Neckar, Germany) that was calibrated in advance.

During experiment (B), the PAWP was maintained at 25 cmH_2_O while the CP was decreased from 35 to 15 cmH_2_O in decrements of 5 cmH_2_O. We defined the leak CP (LCP) to be the highest CP at which leakage was detected. When leakage was not detected, the lowest CP studied was considered the LCP. On the basis of the above definitions, improvement of the air-sealing performance will result in a higher LAWP and a lower LCP. We compared the incidence of air leakage between the three different treatment groups by Fisher’s exact test and compared LAWP and LCP between the treatment groups by Kruskal-Wallis test and Dunn’s multiple comparison test. A *p* value of 0.05 was considered to indicate statistical significance.

## Results

The incidences of air leakage during experiments (A) and (B) are shown in Tables 1, 2. We detected air leakage in most of the tubes in the None and Water groups in both experiments. The incidence of leakage in the KY group was significantly lower than that in the other two groups for all conditions in experiment A using tracheal tubes with IDs of both 4.0 and 5.0 mm. For experiment B, the KY group showed a significantly lower incidence of leakage than the other two groups in most of the conditions for ID 4.0 mm and in all conditions for ID 5.0 mm. The difference did not reach statistical significance in one of the conditions for ID 4.0 mm.

**Table 1:** The incidence of air leakage in experiment A

		Peak airway pressure (cmH_2_O)				
Tube size	Lubrication	5	10	15	20	25
4.0 mm	None	5/5	5/5	5/5	5/5	5/5
	Water	3/5	5/5	5/5	5/5	5/5
	K-Y jelly	0/5	0/5	0/5	0/5	1/5
	*P* value	0.009	<0.001	<0.001	<0.001	0.011
5.0 mm	None	10/10	10/10	10/10	10/10	10/10
	Water	10/10	10/10	10/10	10/10	10/10
	K-Y jelly	1/10	1/10	2/10	3/10	3/10
	*P* value	<0.001	<0.001	<0.001	<0.001	<0.001

The cuff pressure was maintained at 20 cmH_2_O while the peak airway pressure was varied from 5 to 25 cmH_2_O in increments of 5 cmH_2_O

**Table 2:** The incidence of air leakage in experiment B

		Cuff pressure (cmH_2_O)				
Tube size	Lubrication	15	20	25	30	35
4.0 mm	None	5/5	5/5	5/5	5/5	5/5
	Water	4/5	4/5	4/5	4/5	4/5
	K-Y jelly	1/5	0/5	0/5	0/5	0/5
	*P* value	0.051	0.006	0.006	0.006	0.006
5.0 mm	None	10/10	10/10	10/10	10/10	10/10
	Water	9/10	9/10	9/10	9/10	9/10
	K-Y jelly	2/10	1/10	1/10	1/10	1/10
	*P* value	<0.001	<0.001	<0.001	<0.001	<0.001

The peak airway pressure was maintained at 25 cmH_2_O while the cuff pressure was varied from 35 to 15 cmH_2_O in decrements of 5 cmH_2_O

As shown in Table 3, for both ID 4.0 mm and 5.0 mm, the KY group showed significantly higher LAWP and significantly lower LCP compared with the other two groups. There was no significant difference in air-sealing performance between the None and Water groups.

**Table 3:** Leak airway pressure and leak cuff pressure in three different lubrication conditions

		Lubrication		
Tube size	Pressures	None	Water	K-Y jelly
4.0 mm	LAWP (cmH_2_O)	5 (5)	5 (5)	25 (5–25)**,^##^
	LCP (cmH_2_O)	35 (35)	35 (15–35)	15 (15)**,^#^
5.0 mm	LAWP (cmH_2_O)	5 (5)	5 (5)	25 (15–25)***,^###^
	LCP (cmH_2_O)	35 (35)	35 (15–35)	15 (15–35)***,^###^

LAWP: leak airway pressure, defined as the lowest peak airway pressure at which air leakage was detected. LCP: leak cuff pressure, defined as the highest cuff pressure at which air leakage was detected. When leakage was not detected, the highest peak airway pressure or the lowest cuff pressure studied was defined as LAWP or LCP, respectively

The values are reported as median and (range). *N* = 5 for the experiments with ID 40 mm and *N* = 10 for those with ID 5.0 mm

*p < 0.05 vs. None group. **p < 0.01 vs. None group. ***p < 0.001 vs. None group. #*p* < 0.05 vs. Water group. ##*p* < 0.01 vs. Water group. ###*p* < 0.001 vs, Water group

## Discussion

Lubrication with K-Y jelly improved the air-sealing performance of the pediatric cuffed tracheal tubes with ID 4.0 and 5.0 mm in our model experiment. Lubrication of the cylindrical shaped PVC cuff with K-Y jelly significantly lowered the CP needed to prevent air leakage compared with the non-treated and water-treated conditions. The application of water had no effect on the air-sealing properties compared with non-treated condition.

The outer diameters of the studied tracheal tubes with ID 4.0 and 5.0 mm were 5.6 and 6.7 mm, and the diameters of their cuffs reached 13 mm and 16 mm at an inflation pressure of 20 cmH_2_O, respectively. Therefore, when we inflated the cylindrical shaped PVC cuff in the tracheal models with the diameters of 9 and 12 mm, we observed longitudinal folds formed on the cuff surface, as we predicted. We also observed that KY jelly partially filled the space between the cuff surface and the wall of the model trachea. It is rational to assume that K-Y jelly prevents both air and liquid leakage through the channels formed by these longitudinal folds irrespective of differences in physical and rheological conditions. According to an earlier report, lubrication with K-Y jelly completely prevented liquid leakage in a model study using adult tracheal tubes with a conventional PVC cuff [4]; however, prevention of air leakage by K-Y jelly was incomplete in our study. These differences in the effects of lubrication may be explained by differences in various conditions between the two studies, such as brands of tracheal tubes used, the viscosity of air and water, and the pressure gradient inducing leakage. Our results may also suggest that KY jelly may not be optimal for the prevention of air leakage. It is likely that KY jelly is ineffective when large longitudinal folds are formed and that the effects of the lubricant last only for a short time. We should take these problems into account and consider other methods for preventing air leakage such as continuous control of CP in situations requiring long periods of ventilation.

We chose the fixed CP of 20 cmH_2_O for experiment A. For adult patients, the safe range of the CP is variously considered to be 20 to 30 cmH_2_O or 22 to 32 cmH_2_O. [14, 15] The safe CP for pediatric patients is assumed to be lower than these values because of the assumed lower perfusion pressure in the tracheal wall, but it is uncertain. Earlier studies showed that cuffed endotracheal tubes resulted in comparable post-extubation airway morbidity as uncuffed tubes when the CP was limited to 25 mmHg in young children ranging in age from 0 to 8 years (mean, 3.3 years) and to 20 cmH_2_O in pediatric patients ranging in age from 0 to 5 years (median, 1.9 years). [16, 17] Therefore, we considered that 20 cmH_2_O would be in the safe range for children in the trachea simulated in our study.

We chose the fixed PAWP of 25 cmH_2_O for experiment B because we assumed that a PAWP of 25 cmH_2_O is the upper limit encountered in clinical practice. It is common to confirm air leakage around a tracheal tube by manually applying positive airway pressure of 20–30 cmH_2_O in pediatric anesthesia practice. [18] We consider that the condition of no air leakage at 25 cmH_2_O PAWP would be safe as long as the baseline CP is in the safe range when tracheal tubes with large-volume cuffs are used. Therefore, we chose 25 cmH_2_O as the maximum PAWP in this study. We also demonstrated that K-Y jelly improved air-sealing performance at the CP of 15 cmH_2_O in experiment B and at the lower PAWP in experiment A (Tables 1, 2).

Good air sealing provides several benefits in pediatric patients. These include effective ventilation, enhanced reliability of end-tidal gas monitoring and measurements of pulmonary mechanics, diminished contamination of the environment, less waste of inhalation anesthetics, and allowance of the use of low-flow anesthesia. [16, 17] Therefore, we think that there is an increasing need for safe cuffed endotracheal tubes with good air sealing for pediatric patients as cost concerns regarding the consumption of inhalation anesthetics increase. The Microcuff endotracheal tube seems to meet this need perfectly because it offers very good sealing to prevent air and liquid leakage at a very low CP that avoids stress on the tracheal wall. [1, 6, 19] However, a drawback of this product is its high price. Our results suggest that lubrication with K-Y jelly would improve the air-sealing performance of pediatric tracheal tubes with a conventional PVC cuff and render these inexpensive tubes safe in providing a good air seal.

There are several limitations in our study. First, this study was performed with a tracheal model. The effectiveness of lubrication may be affected by various properties of the human trachea, such as the presence of humidity and airway secretions, the elasticity of the tracheal wall, and the irregular surface of the tracheal mucosa. Our findings need to be verified in clinical settings. Furthermore, the duration of the improvement in air-sealing performance in vivo should also be examined. A clinical study demonstrated that preventive effects of lubrication with KY jelly on liquid leaks lasted about 24 h in adult patients intubated with cuffed tracheostomy tubes. [3] To our knowledge, however, no study has investigated air leakage. Second, we only used two sizes of tracheal tube sized appropriately for older children. Further studies are needed to clarify whether our results will hold in other conditions such as simulating younger patients and using different tracheal tubes. Third, we did not evaluate the magnitudes of leakage because of the limited accuracy of the flow and volume measurements in our experiments, and this obscured the significance of the leakage observed. Finally, risks and benefits of applying lubricants need to be evaluated in various patient age groups as it has been shown that excess lubrication might obstruct the distal opening of the tracheal tube in infants. [20]

## Conclusions

Lubrication with K-Y jelly improved the air-sealing performance of a pediatric endotracheal tube with a cylindrical shaped PVC cuff in our model experiments. Lubrication with K-Y jelly but not water significantly lowered the CP needed to prevent air leakage. Future clinical studies are warranted to verify our findings and to evaluate the safety of using lubricants in various patient age groups.

## Abbreviations

CP: Cuff pressure
CTT: Cuffed tracheal tube
ID: Internal diameter
LAWP: Leak airway pressure
LCP: Leak cuff pressure
PAWP: Peak airway pressure
PVC: Polyvinyl chloride
